# Supplementary material for: Incorporation of an invasive plant into a native insect herbivore food web
Source: PeerJ. 2016 May 10;4:e1954. doi: 10.7717/peerj.1954 (PMC4867706; doi:10.7717/peerj.1954)
Supplement: Text S1 [file peerj-04-1954-s001.docx]

Structure analysis on all data (inclusive 4 loci not adhering to Hardy-Weinberg equilibrium)


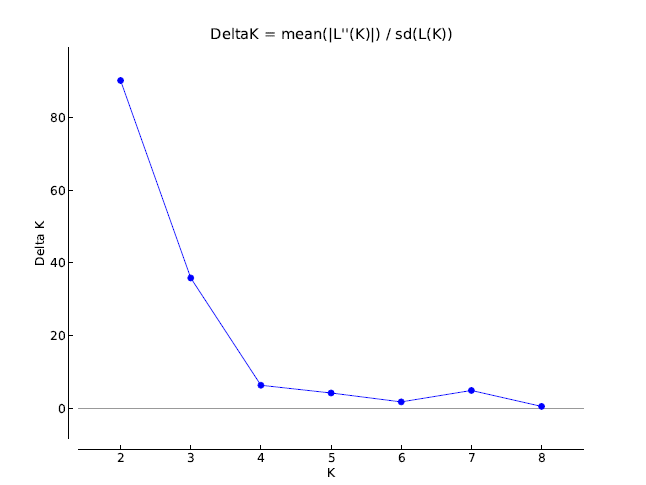


The graph shows that the number of groups is 2 (or 1).

Genotype assignments under K=2. Groups of individuals have been ordered by locality and then host. In other words, the first half of the individuals are from one host, the second half from the other. We conclude that host does not affect population stucture.


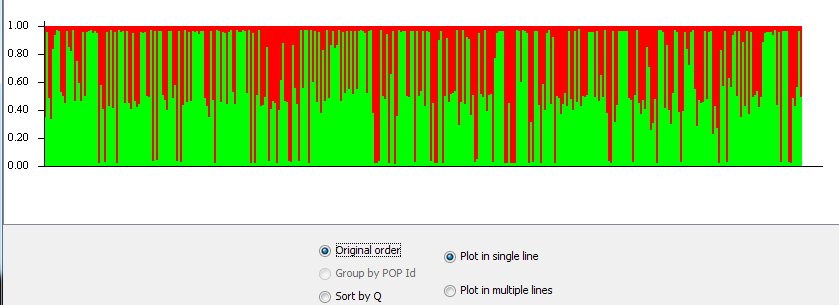


AMOVA

Source of Sum of Variance Percentage

variation d.f. squares components of variation

----------------------------------------------------------------------

Among

Sampling sites 4 335.291 0.37826 Va 1.69 **

Among

Sampling sites

within

hosts 4 119.813 0.09129 Vb 0.41 ***

Within

populations 749 16375.987 21.86380 Vc 97.90

----------------------------------------------------------------------

Total 757 16831.091 22.33335

Differentiation between sampling sites (considering the beetles on the different hosts as one )

----------------------------------------------------------------------

Source of Sum of Variance Percentage

variation d.f. squares components of variation

----------------------------------------------------------------------

Among

populations 4 316.580 0.39451 Va 1.76***

Within

populations 753 16535.560 21.95957 Vb 98.24

----------------------------------------------------------------------

Total 757 16852.140 22.35409

----------------------------------------------------------------------

Fixation Index FST : 0.01765

----------------------------------------------------------------------
