# Supplementary material for: Incorporation of an invasive plant into a native insect herbivore food web
Source: PeerJ. 2016 May 10;4:e1954. doi: 10.7717/peerj.1954 (PMC4867706; doi:10.7717/peerj.1954)

To be able to compare our (dry leaf weight) results with fresh weight data from the literature, we weighed 10 *P. serotina* and 10 *P. padus* leaves after collecting. They were subsequently dried for 48 h at 105°C in an oven and then weighed again. The results (see below) were used to obtain wet-to-dry weight conversion factors of 0.35 for *P. padus* and 0.36 for *P. serotina*.

We compared our data with those of Santamour (1998) as follows. Given that 1 mol HCN is produced by the degradation of 1 mol glycoside (Swain et al., 1991), molecular weights for prunasin and amygdalin are 295 and 457, respectively, and their mean ratio in both *Prunus* species in Santamour’s study is 22:1, then Santamour’s HCN levels derive from 29.6 mg cyanogenic glycosides per g fresh leaf material in North America.


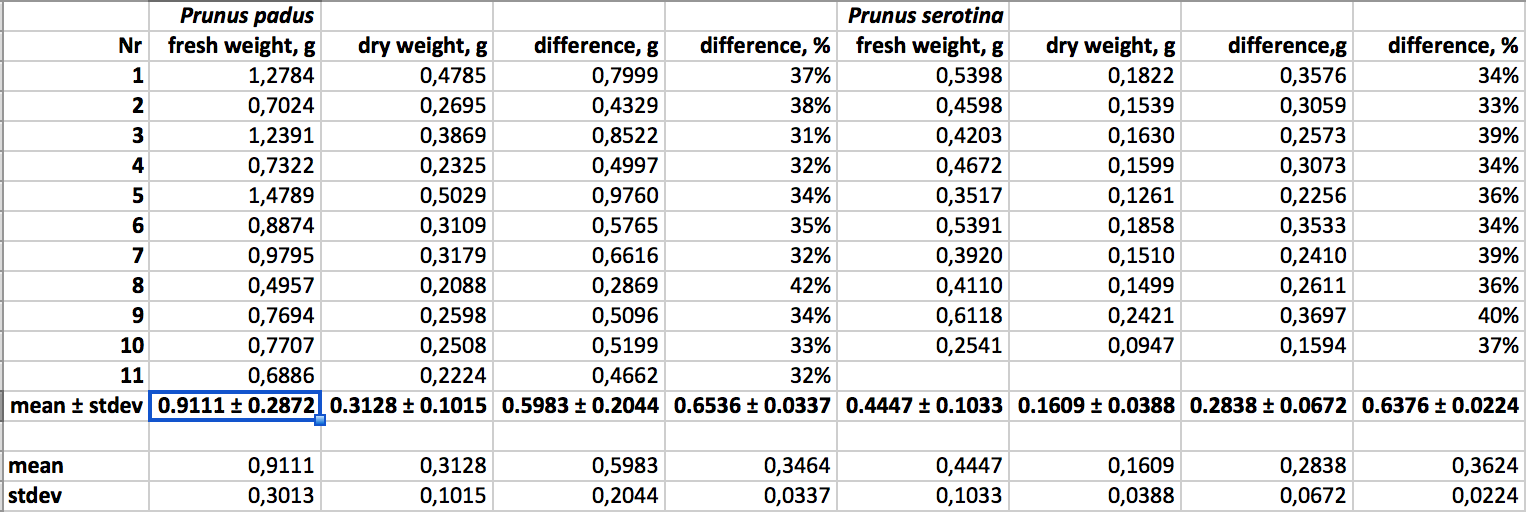

Supplement: Text S2 [file peerj-04-1954-s002.docx]
