# Supplementary material for: Incorporation of an invasive plant into a native insect herbivore food web
Source: PeerJ. 2016 May 10;4:e1954. doi: 10.7717/peerj.1954 (PMC4867706; doi:10.7717/peerj.1954)
Supplement: Table S6 [file peerj-04-1954-s008.docx]

**Table S6**

**SNP Loci that showed indications of genetic differentiation between both host plants in the leaf beetle *Gonioctena quinquepunctata*.**

locus name^1^ SNP^2^ Allele freq. *Prunus* (N)^3^ Allele freq. *Sorbus* (N)^3^ *P* [AMOVA]^4^

72443613_231 A / T 0.62 / 0.38 (318) 0.44 / 0.56 (370) <0.05

72370850_384 A / G 0.39 / 0.61 (346) 0.29 / 0.71 (412) <0.05

72300691_711 C / T 0.56 / 0.44 (332) 0.43 / 0.57 (394) N.S.

72240532_350 A / G 0.31 / 0.69 (300) 0.46 / 0.54 (326) N.S.

72446378_229 C / G 0.73 / 0.27 (344) 0.83 / 0.17 (412) N.S.

72253142_689 A / G 0.75 / 0.25 (322) 0.66 / 0.34 (380) N.S.

72448184_218 C / T 0.53 / 0.47 (346) 0.63 / 0.37 (412) N.S.

72453577_723 A / T 0.32 / 0.68 (338) 0.24 / 0.76 (400) N.S.

^1^ [contig number]_[nucleotide position]

^2^ alternative nucleotides

^3^ N = number of alleles sampled in the population

^4^ after strict Bonferroni correction for multiple tests
