# Supplementary material for: Incorporation of an invasive plant into a native insect herbivore food web
Source: PeerJ. 2016 May 10;4:e1954. doi: 10.7717/peerj.1954 (PMC4867706; doi:10.7717/peerj.1954)
Supplement: Table S7 [file peerj-04-1954-s009.docx]

Results of statistical tests on host plant preference in *Gonioctena quinquepunctata*

**Presence or absence of preference (proportion test, tested against 0.5)**

**n chi^2^  d.f. *p***

all data combined 341 32.950 1 <0.0001

adults + larvae from *Sorbus* 221 33.466 1 <0.0001

--adults 83 21.253 1 <0.0001

--larvae 138 13.399 1 0.0003

adults + larvae from *Prunus* 120 3.008 1 0.0828

--adults 63 1.587 1 0.2077

--larvae 57 1.123 1 0.2893

**Difference in preference**

**n LRT d.f. *p***

original host x life stage 341 0.009 1 0.9264

original host 341 4.855 1 0.0276

life stage 341 1.258 1 0.2620

date 341 4.733 1 0.0296

collection location 341 2.908 1 0.5734

cage 341 9.411 1 0.0022
