# Supplementary material for: Incorporation of an invasive plant into a native insect herbivore food web
Source: PeerJ. 2016 May 10;4:e1954. doi: 10.7717/peerj.1954 (PMC4867706; doi:10.7717/peerj.1954)

**Table S8:**

**summary tables of general information on response and explanatory variables from entire dataset.**


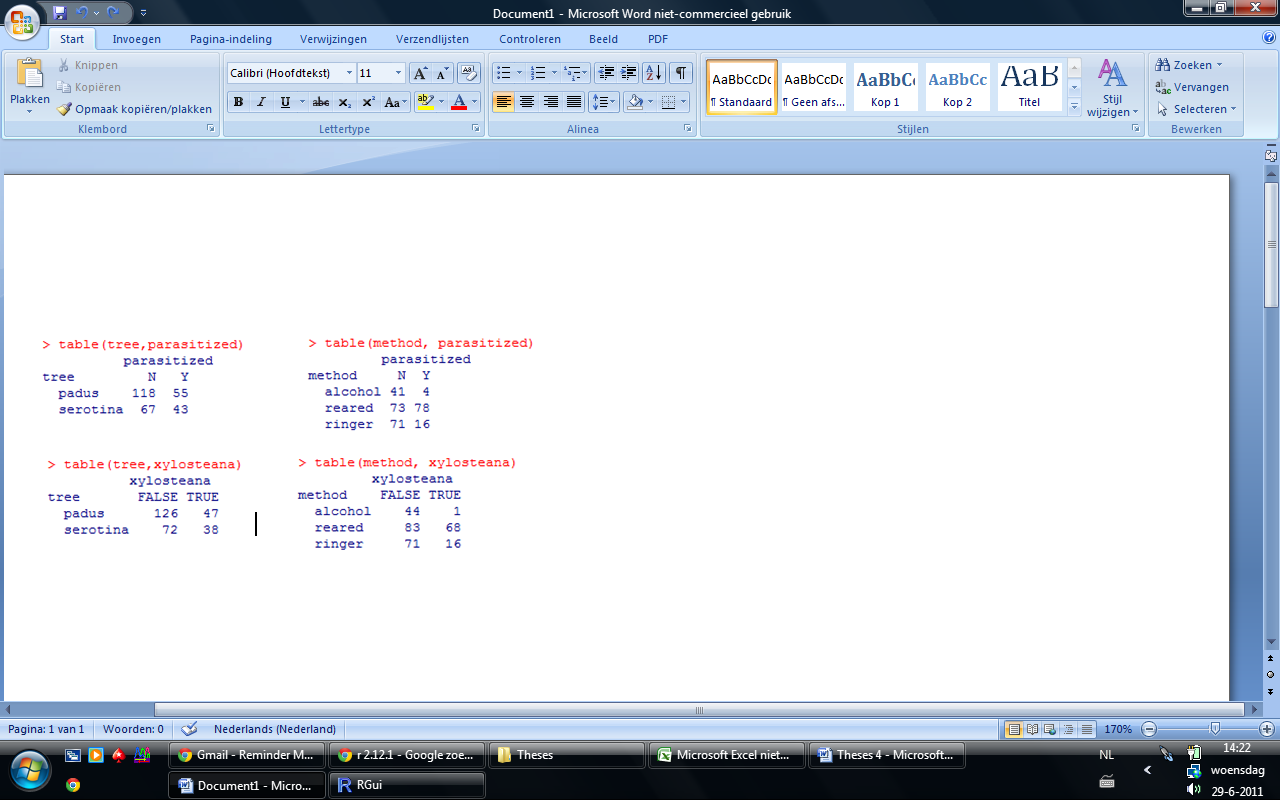

Supplement: Table S8 [file peerj-04-1954-s010.docx]
